# Early Detection of Blast-Induced Hidden Hearing Loss in Military Personnel Using an Extended Audiological Battery

**DOI:** 10.3390/diagnostics16182986

**Published:** 2026-09-15

**Authors:** Kuan-Yu Chen, Tzu-Ching Chou, Ting-Li Hung, Hsin-Chien Chen

**Affiliations:** 1 Department of Otolaryngology-Head and Neck Surgery, Tri-Service General Hospital, National Defense Medical University, Taipei 11490, Taiwan; luke840818@gmail.com (K.-Y.C.); andrea860208@gmail.com (T.-C.C.); tinglee1230@gmail.com (T.-L.H.); 2 Department of Otolaryngology-Head and Neck Surgery, School of Medicine, College of Medicine, National Defense Medical University, Taipei 11490, Taiwan

**Keywords:** acoustic trauma, blast injury, hearing loss, hidden hearing loss, extended high-frequency audiometry, tympanic membrane perforation

## Abstract

**Background/Objectives**: Military personnel are frequently exposed to high-risk acoustic trauma from blast waves. While macroscopic injuries like tympanic membrane perforation (TMP) are easily identified, subclinical microscopic damage, such as hidden hearing loss (HHL), often goes undetected by standard audiometry. This study investigates the auditory impacts of an accidental ammunition depot explosion, focusing on the diagnostic value of extended high-frequency (EHF) audiometry and objective electrophysiological measures in detecting early-stage blast-induced damage. **Methods**: This retrospective case series included eight military personnel involved in a 120 mm howitzer shell explosion at an ammunition depot. Subjects were categorized into direct blast exposure (*n* = 2) and indirect impact (*n* = 6). Audiological evaluations included otoscopy, standard pure-tone audiometry (0.25–8 kHz), EHF audiometry (9–14 kHz), auditory brainstem response (ABR), and distortion product otoacoustic emissions (DPOAEs). Results were compared to reference and control groups. Two patients with direct trauma underwent type I tympanoplasty to repair TMP. **Results**: Directly exposed personnel sustained near-total TMPs and significant mixed hearing loss. In contrast, the six indirectly impacted personnel exhibited “clinically normal” standard audiograms; however, their EHF thresholds at 9, 10, 12.5, and 14 kHz were significantly elevated compared to controls (*p* < 0.05). DPOAE amplitudes at 1, 1.5, and 10 kHz were significantly reduced in the blast group, indicating outer hair cell dysfunction. ABR showed a prolonged wave I latency with statistical significance (*p* = 0.014), but a decreased wave I amplitude was nonsignificant between groups; these findings are suggestive of possible cochlear synaptopathy, consistent with, but not diagnostic of, HHL. **Conclusions**: Blast exposure may produce both overt and subclinical auditory injury. Even in individuals with normal conventional audiograms, our preliminary findings suggest that EHF audiometry and otoacoustic emission testing show promise in identifying early cochlear dysfunction consistent with HHL. While further validation in larger cohorts is required to establish definitive diagnostic utility, these hypothesis-generating observations highlight the potential value of advanced audiologic assessments for the detection and monitoring of blast-related auditory injury in military personnel.

## 1. Introduction

Military personnel represent a high-risk population for hearing loss. The auditory system is particularly vulnerable to explosive events because it is designed to detect pressure waves and may therefore be disproportionately affected by the intense energy generated by blast overpressure [1]. Acoustic trauma refers to a clinical condition characterized by persistent hearing loss that occurs immediately after exposure to impulse noise or blast waves [2]. Otologic injury is typically classified as a primary blast injury, and common causes in military settings include exposure to high-intensity impulse noise from weapons firing or explosive detonations.

Blast exposure produces rapid over-pressurization of air molecules, generating impulse noise characterized by extremely high peak pressure levels. Such exposure can lead to immediate sensorineural, conductive, or mixed hearing loss [3]. A blast occurs when solids or liquids are rapidly converted into gas, producing a sudden expansion of pressurized gas that propagates through the surrounding medium [4]. This process generates a peak pressure wave, known as a shock wave, followed by a rapid drop in atmospheric pressure (under-pressurization) and a high-velocity blast wind [3].

Individuals experiencing acoustic trauma may develop both macroscopic and microscopic otologic injuries. Macroscopic damage includes injury to the external auditory canal, tympanic membrane hemorrhage or perforation (tympanic membrane perforation, TMP), and disruption of the ossicular chain. Microscopic injury may involve perilymphatic fistula, cochlear injury, or subtle auditory dysfunction such as hidden hearing loss (HHL). HHL is a recently recognized auditory disorder characterized by impaired auditory neural processing despite normal conventional audiometric thresholds, particularly affecting hearing in noisy environments [5]. This condition may be detectable using extended high-frequency (EHF) audiometry. The proposed mechanisms of HHL include cochlear synaptopathy, resulting from the loss of synaptic connections between inner hair cells and auditory nerve fibers [5]. Additional proposed mechanisms include cochlear demyelination and mild or persistent hair cell dysfunction [6,7].

Most studies of blast-related otologic injury have focused on the prevalence and healing rate of TMP, associated tinnitus, and the patterns of hearing loss detected using standard audiometry. In contrast, the present study evaluates not only macroscopic otologic injuries, but also explores the potential mechanisms underlying HHL in military personnel exposed to a blast event.

## 2. Materials and Methods

**Figure 1:**
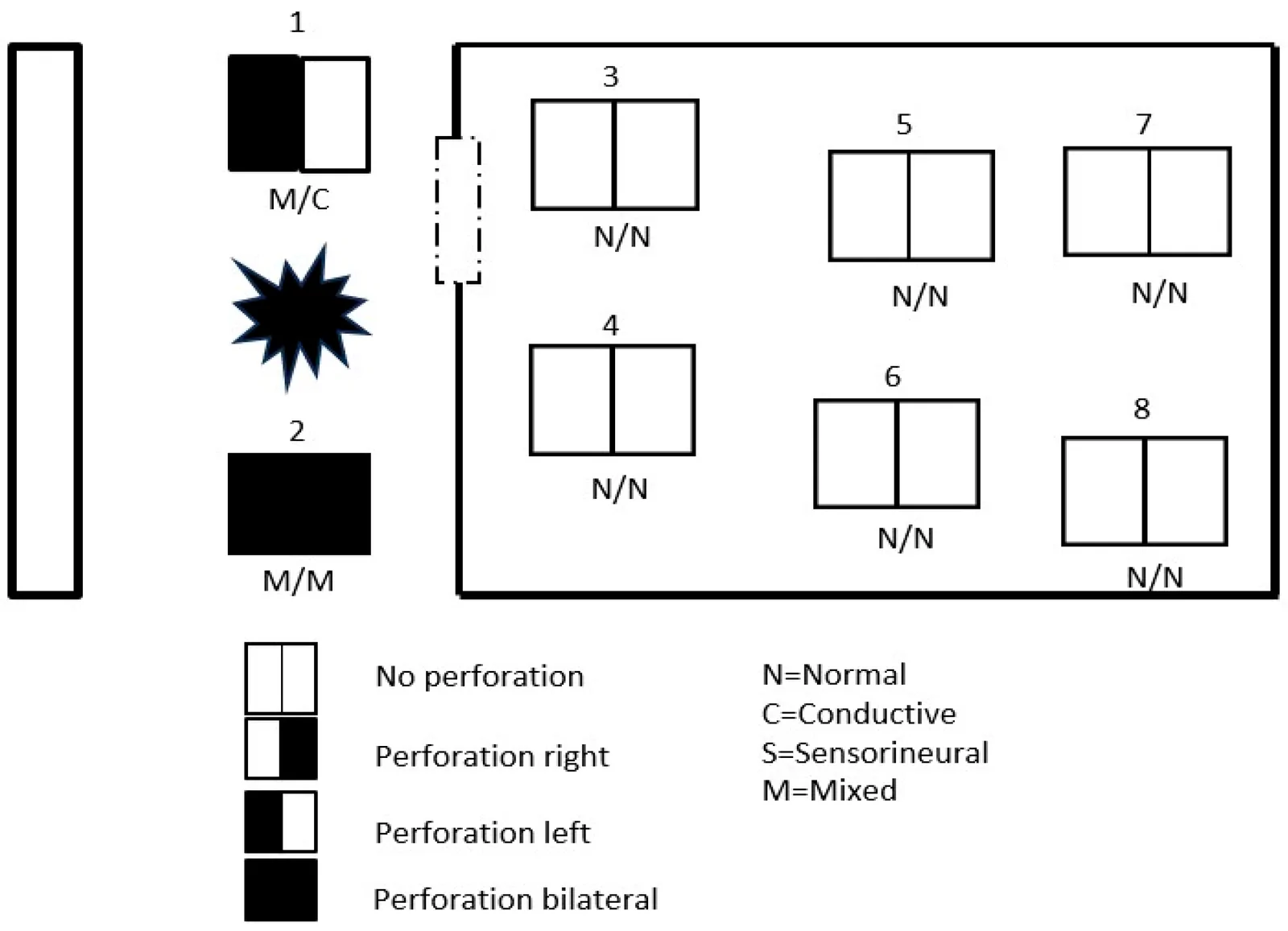
Approximate locations of military personnel located near the ammunition depot explosion, with the findings of tympanic membrane and the type of hearing loss indicated when available. Star symbol represents the location of the bomb.

This retrospective case series included eight military personnel affected by a 2023 ammunition depot explosion who were treated at Tri-Service General Hospital in Taiwan (Figure 1). Two patients sustained severe blast injuries with traumatic limb loss. The remaining six personnel, who were located in an adjacent building approximately 5 m from the explosion site and had no concomitant extremity trauma, underwent serial audiologic evaluations.

Patients 1–8 comprised young male and female adults aged 20–40 years with no prior history of otologic or audiologic disorders in their institutional medical records. Patients 1 and 2 underwent initial audiometric testing once hemodynamic stability was achieved. For patients 3–8, a comprehensive battery of routine and advanced audiological evaluations (Table 1), distortion product otoacoustic emissions (DPOAEs, Table 2) and auditory brainstem response (ABR, Table 3) were performed 1 day post-explosion; for these patients, transient tinnitus and vertigo subsided within a few days after the blast incident. The control group was matched with six healthy people aged 20–30 years from our institution, all of whom had no documented history of trauma or hearing impairment in their medical records and underwent DPOAE and ABR evaluations. To avoid selection bias, the reference group was established based on the normative mean audiometric data for the 25–34-year-old cohort in a military environment cited from Škerková et al. [8].

Physical and otoscopic examinations were conducted to initially assess physical trauma to the external auditory canal and the tympanic membrane. These examinations specifically identified the presence of macroscopic tympanic membrane hemorrhage or TMP and estimated the percentage of the perforated area relative to the entire tympanic membrane. None of the eight patients in the blast-exposed cohort received systemic (oral or intravenous) or intratympanic corticosteroid therapy following the incident.

### 2.1. Audiological Assessment

Conventional hearing thresholds from 250 to 8000 Hz (8 kHz) were evaluated via standard pure-tone audiometry (PTA) using a Grason-Stadler GSI 61 clinical audiometer (Grason-Stadler, Eden Prairie, MN, USA) equipped with TDH-50P headphones (Telephonics Corporation, Farmingdale, NY, USA) in a sound-attenuated chamber. To detect early subclinical micro-lesions, EHF audiometry was performed to test ultra-high frequencies beyond the conventional range, specifically recording thresholds at 9, 10, 12.5, and 14 kHz. EHF measurements utilized the same GSI 61 audiometer, alternatively equipped with circumaural headphones supporting ultra-high frequencies (Sennheiser HDA 200, Wedemark, Germany).

### 2.2. Distortion Product Otoacoustic Emission Assessment

DPOAEs were utilized to assess the microfunctional status of cochlear outer hair cells (OHCs). Response amplitudes across multiple frequencies were measured, with specific analysis focused on frequencies exhibiting significant declines, namely 1k to 10 kHz. DPOAE testing was performed using the Bio-Logic Scout Sport System (Natus Medical Inc., San Carlos, CA, USA), with acoustic stimuli delivered and emissions recorded via an insert ear canal probe.

### 2.3. Auditory Brainstem Response Assessment

To evaluate auditory nerve integrity and potential cochlear synaptopathy, ABR testing was conducted to measure the latency and amplitude of wave I. ABR traces were obtained using a Bio-Logic Navigator Pro Evoked Potential System (Natus Medical Inc.), utilizing 90 dB nHL click stimuli.

### 2.4. Statistical Analysis

Independent sample t-tests were conducted to compare auditory outcomes between the study groups. The first analysis compared standard PTA thresholds between the exposed and control groups. Subsequent analyses compared DPOAEs and ABR between the exposed and control groups. Additionally, the Kruskal–Wallis test was employed to compare EHF thresholds among the exposed, control, and reference groups. A result is considered statistically significant when the *p*-value is less than 0.05 (*p* < 0.05).

## 3. Results

The explosion occurred directly in front of the ammunition depot and involved the detonation of a 120 mm howitzer shell (Figure 1, left). Two personnel were directly exposed to the blast, whereas six others were located inside an adjacent building and experienced indirect blast effects (Figure 1, right). These individuals were sequentially designated as patients 1 through 8.

### 3.1. Patients 1 and 2: Direct Blast Exposure

Initial audiologic assessments were unavailable for patients 1 and 2 because both patients sustained severe blast injuries requiring intensive care unit management.

**Figure 2:**
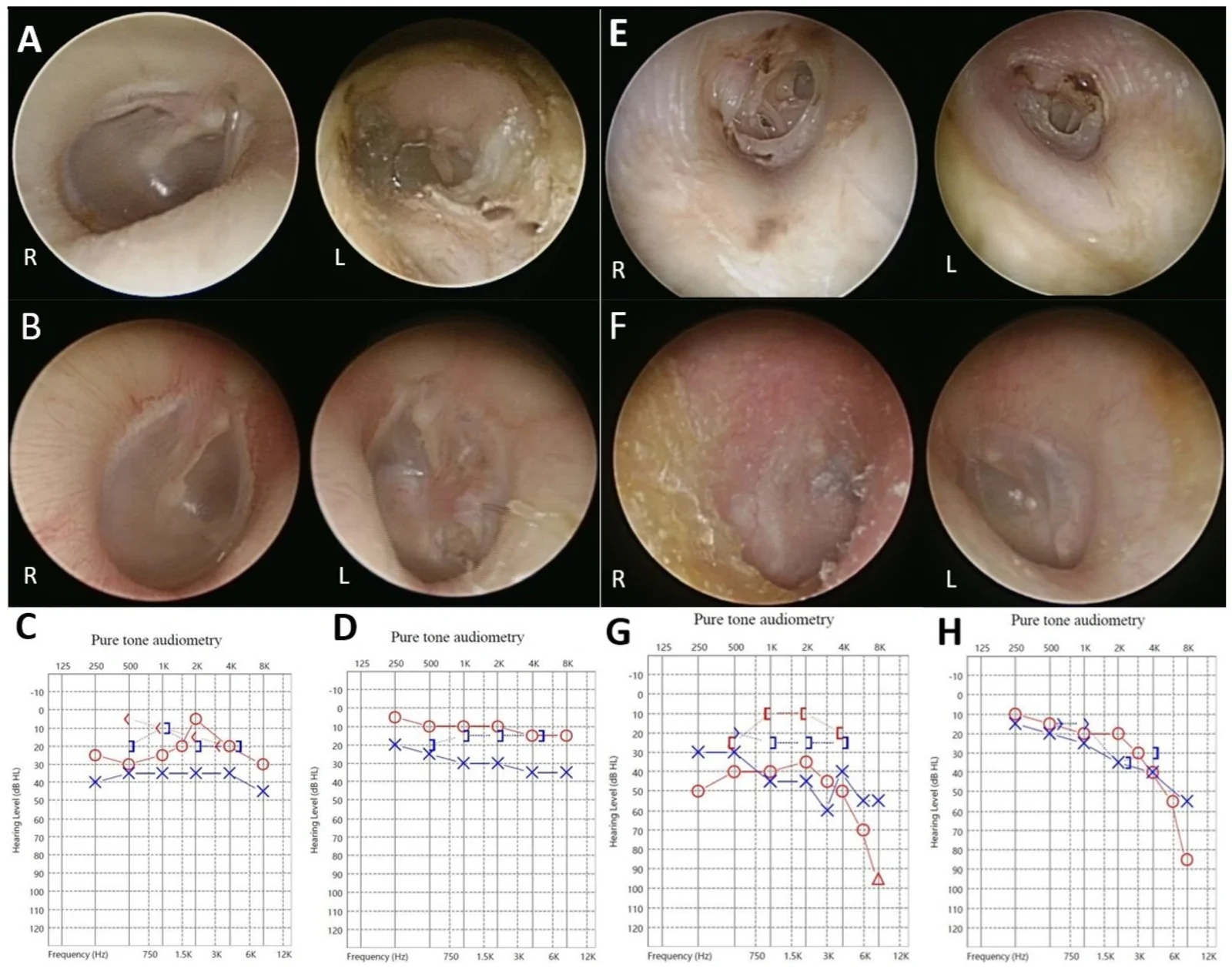
(**A**) Preoperative and (**B**) postoperative otoscopic views of patient 1. (**C**) Preoperative and (**D**) postoperative pure-tone audiograms of patient 1. (**E**) Preoperative and (**F**) postoperative otoscopic views of patient 2. (**G**) Preoperative and (**H**) postoperative pure-tone audiograms of patient 2.

Patient 1 presented with left upper-limb amputation and right lower-limb compartment syndrome. Otoscopic examination revealed a left TMP involving approximately 80% of the eardrum (Figure 2A). Standard PTA performed after stabilization demonstrated bilateral mixed hearing loss with an average air–bone gap (ABG) of approximately 20 dB (Figure 2C). After six months of observation, type I tympanoplasty was performed on the left ear. At 5-month postoperative follow-up, a residual TMP involving approximately 5% of the eardrum remained (Figure 2B). Hearing thresholds improved compared with baseline; however, residual conductive hearing loss persisted in the left ear, with an ABG of approximately 10 dB (Figure 2D).

Patient 2 presented with left wrist amputation and 26% total body surface third-degree burns. Otoscopic examination demonstrated bilateral TMPs involving approximately 50–80% of the tympanic membranes (Figure 2E). Standard PTA performed after stabilization revealed bilateral mixed hearing loss, with an average ABG of approximately 20 dB in both ears (Figure 2G). After seven months of observation, sequential bilateral type I tympanoplasty procedures were performed. At 4-month postoperative follow-up, both tympanic membranes were intact (Figure 2F), and hearing thresholds had improved compared with preoperative levels; however, persistent high-frequency sensorineural hearing loss (SNHL) remained (Figure 2H).

### 3.2. Patients 3–8: Indirect Blast Exposure

**Figure 3:**
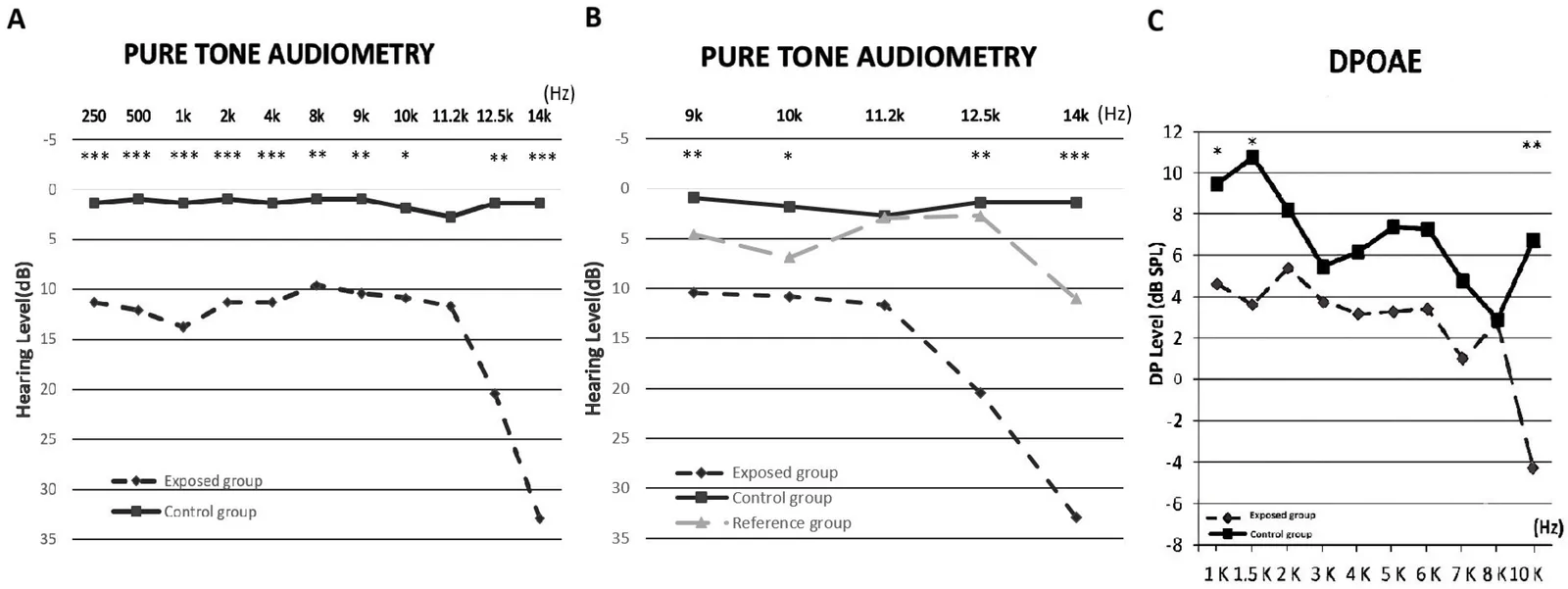
(**A**) Hearing thresholds of exposed group are significantly higher than control group at 250~14k Hz. (**B**) Hearing thresholds of exposed group are significantly higher than reference group and control group at 9k, 10k, 12.5k and 14k Hz. (**C**) At 1k, 1.5k, and 10k Hz, the average DP level in the exposed group was significantly lower than control group. * *p* < 0.05, ** *p* < 0.01, *** *p* < 0.001.

**Figure 4:**
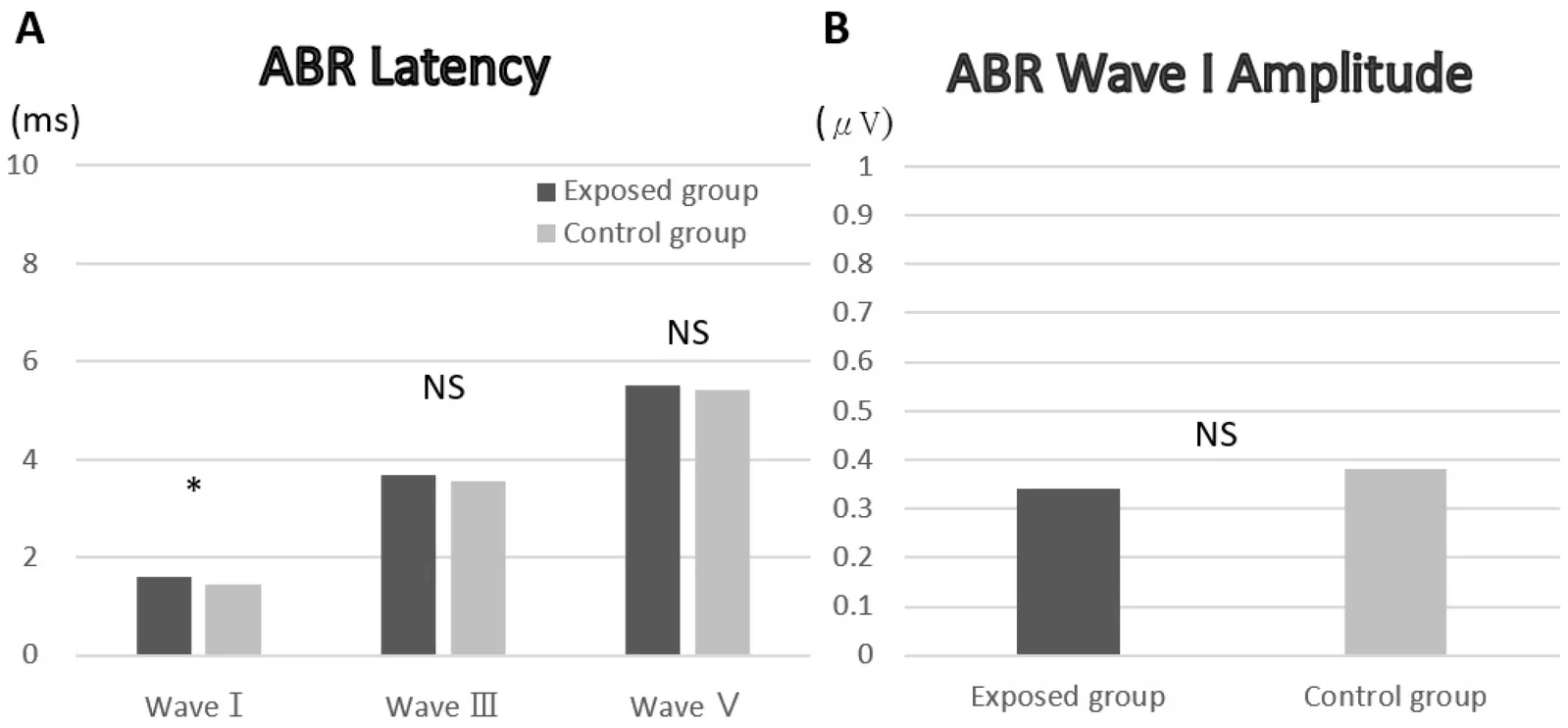
(**A**) ABR showed prolonged latency in waves I, III, V, with a significant difference in only wave I and (**B**) decreased amplitude in wave I in the exposed group, but not statistically significant compared to control group. * *p* < 0.05; NS, not significant.

Patients 3 through 8 were located inside the adjacent building and experienced indirect blast exposure. These six patients (aged 20–40 years, including both males and females) had no recorded history of chronic illness, occupational noise exposure, or prior head/acoustic trauma according to medical records. All patients underwent initial audiological evaluation 1 day post-explosion. All patients reported transient tinnitus and vertigo, which resolved within several days. Standard PTA (250 Hz–8 kHz) revealed normal hearing thresholds, and otoscopic examination showed no TMP. Although standard pure-tone audiometry (PTA) thresholds in the exposed group remained within clinically normal limits, an observable downward shift was noted. Comparison with our institutional control group of six age-matched individuals using *t*-test revealed that this threshold elevation on standard PTA was statistically significant (Table 1 and Figure 3A). Furthermore, to minimize sampling bias, age-matched normative data for the 25–34-year-old cohort from Škerková et al. [8] were adopted for extended high-frequency (EHF) comparisons. Across the three-group comparison, statistically significant threshold elevations were identified at 9, 10, 12.5, and 14 kHz (*p* < 0.05) (Figure 3B). DPOAE amplitudes at 1, 1.5, and 10 kHz were significantly reduced in the exposed group compared to the control group (Table 2 and Figure 3C). In ABR testing, the mean latency of wave I was prolonged by 0.14 ms compared with the control group, with a significant difference (*p* = 0.014), while the mean amplitude of wave I decreased by 0.04 μV with no significance (Table 3 and Figure 4).

## 4. Discussion

According to reports from the Boston Marathon bombing, otologic trauma was the most common physical injury among affected individuals [9]. Proximity to the blast and the presence of significant non-otologic injuries were identified as predictors of TMP [9]. Among patients with TMP following the Boston Marathon bombing, approximately 38% experienced spontaneous healing [9,10]. Other civilian studies investigating blast-induced TMP have reported spontaneous healing rates ranging from 38% to 82% [11,12,13].

In patients 1 and 2, both patients ultimately underwent type I tympanoplasty; however, postoperative air-conduction thresholds did not return to normal levels. Both individuals sustained substantial non-otologic injuries, suggesting exposure to greater blast overpressure. Such intense blast exposure likely resulted not only in large TMPs requiring surgical repair, but also in more severe inner ear injury, including damage to cochlear synapses. This additional cochlear damage may have limited the potential for complete hearing recovery. Although tympanoplasty effectively addresses the conductive component of hearing loss by closing the ABG, blast exposure frequently causes concomitant SNHL due to injury to cochlear hair cells or neural synapses. Consequently, even after successful tympanic membrane repair, residual high-frequency SNHL may hinder full restoration of hearing function.

In the remaining cases, no obvious structural otologic injury was observed, and standard PTA demonstrated normal hearing thresholds. However, EHF audiometry revealed elevated thresholds, suggesting that blast-induced cochlear damage may initially occur at frequencies beyond the detection range of conventional audiometry. The DPOAE findings reflect the functional status of outer hair cells (OHCs). Specifically, the reduction in DPOAE amplitudes at 10 kHz supports the presence of OHC dysfunction in the high-frequency region, even when conventional audiograms appear normal. While low-frequency outer hair cell (OHC) impairment is difficult to reconcile under classical basal-restricted noise trauma models, this apparent discrepancy reflects the unique physical dynamics of blast overpressure [3,4]. Unlike traveling waves from continuous noise, explosive shock waves deliver instantaneous compressive forces through the cochlear capsule, producing diffuse shear stress along the entire basilar membrane and concurrent basal-to-apical degeneration [10,14]. Moreover, the co-occurrence of mid-to-low frequency DPOAE reduction and elevated extended high-frequency (EHF) thresholds aligns with evidence that EHF loss often indicates generalized subclinical cochlear injury [15]. Thus, the observed mid-to-low frequency OHC deficits in patients 3–8 likely reflect diffuse microscopic barotrauma that compromises fine frequency selectivity despite normal conventional audiometric thresholds.

Although ABR wave I amplitude is often used as a surrogate marker of auditory nerve integrity, no statistically significant reduction was observed in our cohort. This finding contrasts with the classical findings in animal models of HHL, which typically demonstrate reduced wave I amplitudes [5]. This discrepancy highlights the complexity of diagnosing HHL in humans and suggests that electrophysiologic markers derived from animal studies may not directly translate to clinical populations.

Collectively, our findings suggest that acute acoustic trauma predominantly affects high-frequency hearing. Such deficits may remain undetected using conventional audiometry but can be identified using EHF audiometry. Furthermore, the combination of prolonged ABR wave I latency, reduced DPOAE amplitudes, and elevated EHF thresholds may indicate the presence of subclinical auditory dysfunction consistent with HHL, even in individuals with normal hearing.

The pathophysiology of HHL is thought to involve both OHC dysfunction and cochlear synaptopathy. Initially, HHL was primarily attributed to synaptopathy, characterized by the loss of synaptic connections between inner hair cells and auditory nerve fibers. However, more recent studies suggest that subtle OHC damage may also contribute significantly to auditory deficits, particularly in challenging listening environments [5]. Research by Ryan Hoben et al. demonstrated that individuals with hearing thresholds within the “normal” range (≤25 dB HL), particularly those between 15 and 25 dB HL, already exhibit significant reductions in DPOAE amplitudes [7]. These findings suggest that early OHC damage may exist even when hearing thresholds remain within clinically normal limits. Although such damage may not meet the conventional diagnostic criteria for hearing loss, it can still lead to functional impairment, particularly in speech-in-noise perception.

Several mechanisms have been proposed to explain the relationship between OHC dysfunction and auditory performance:

(1) Loss of active cochlear amplification: OHCs provide active amplification and fine tuning of basilar membrane vibrations. When OHC function is compromised, cochlear frequency selectivity deteriorates, impairing auditory filtering [16].
(2) Broadening of neural tuning curves: OHC dysfunction leads to broader auditory nerve fiber tuning curves, reducing frequency selectivity and potentially increasing cross-frequency interference [16,17].
(3) Reduced neural coding resolution: Because broadened tuning curves recruit larger populations of auditory nerve fibers, neural responses lose frequency specificity. This reduction in coding precision impairs the ability to resolve complex acoustic signals, particularly speech in noisy environments [7].

Consistent with these mechanisms, individuals with reduced DPOAE amplitudes or elevated high-frequency thresholds often demonstrate poorer speech perception in background noise [11]. In the present study, clinically normal-hearing subjects (patients 3–8) exhibited significant reductions in DPOAE amplitudes at 1, 1.5, and 10 kHz, supporting the presence of OHC dysfunction. Furthermore, EHF hearing sensitivity (>8 kHz) is highly indicative of the functional status of OHCs in the basal turn of the cochlea. Previous studies have similarly reported lower DPOAE amplitudes at 8 and 10 kHz in individuals exposed to high noise levels, where EHF hearing loss frequently coexists with OHC dysfunction [18].

The most well-documented mechanism underlying HHL is cochlear ribbon synapse degeneration, commonly referred to as cochlear synaptopathy [5]. These synapses are located at the basal pole of inner hair cells and consist of a presynaptic ribbon structure containing neurotransmitter vesicles and a postsynaptic zone containing AMPA-type glutamate receptors [19]. Synaptopathy is commonly induced by noise exposure or aging, particularly following noise levels sufficient to produce a temporary threshold shift. Excessive acoustic stimulation leads to glutamate excitotoxicity within the synaptic cleft, resulting in swelling and degeneration of postsynaptic auditory nerve terminals. Importantly, this pathological process preferentially affects low-spontaneous-rate (LSR) auditory nerve fibers [20,21]. LSR fibers have higher activation thresholds and play a critical role in encoding sound in high-intensity environments while maintaining signal clarity in the presence of background noise. Their selective loss therefore contributes directly to the hallmark symptom of HHL—difficulty understanding speech in noisy environments. In contrast, high-spontaneous-rate fibers, which primarily encode low-intensity sounds and determine hearing thresholds, are generally preserved, explaining why affected individuals often demonstrate normal results on standard PTA [21].

In mouse models, the classic electrophysiological signature of cochlear synaptopathy is a significant reduction in ABR wave I amplitude, while latencies and hearing thresholds remain unchanged [22]. However, the relationship between HHL and ABR wave I amplitude in humans remains controversial, a finding consistent with the observations in this study [5]. Human ABR measurements are influenced by numerous non-pathological variables, including skull thickness, electrode placement, and impedance, resulting in substantial inter-subject variability [5,7,18]. Consequently, ABR wave I alone may not provide sufficient diagnostic precision for identifying synaptopathy in individual patients [5]. Although EHF hearing loss primarily reflects damage to hair cells within the basal turn of the cochlea, such injury is often accompanied by progressive neural degeneration. Moreover, EHF deficits have been associated with accelerated auditory aging, suggesting that they may serve as an early clinical indicator of cochlear vulnerability.

By incorporating EHF audiometry, clinicians may be able to detect auditory dysfunction that would otherwise remain “hidden.” In the context of OHC dysfunction, EHF audiometry provides a direct assessment of cochlear damage localized to the basal turn. In the context of suspected subclinical auditory dysfunction, EHF deficits may serve as a sensitive biomarker of prior acoustic stress, reflecting subclinical OHC vulnerability and indicating that the cochlea has been exposed to blast levels that may pose a risk for subsequent or concurrent neural injury.

Although corticosteroids were not administered post-exposure in our cohort, these agents confer well-documented neuroprotective and anti-inflammatory benefits in traumatic inner ear injury and sudden SNHL [23,24]. Mechanistically, corticosteroids—particularly dexamethasone—mitigate acoustic insult by suppressing proinflammatory cytokine cascades (e.g., TNF-α), preserving Organ of Corti hair cells, and supporting neural transmission fidelity, which has been linked to improved postoperative speech discrimination [23]. Although targeted corticosteroid therapy represents a possible approach to attenuating acute EHF deficits, its efficacy remains theoretical in future study. Future prospective trials are needed to clarify therapeutic windows and define clinical guidelines.

This study has several limitations. First, the sample size was small, because the analysis was based on a single blast event, and may reduce the statistical power of comparisons. Second, the retrospective design limited the availability of baseline audiologic data prior to blast exposure. Finally, although EHF audiometry and DPOAEs suggested early cochlear dysfunction, these findings cannot definitively confirm HHL. Larger prospective studies are needed to further clarify the relationship between blast exposure and subclinical cochlear injury.

## 5. Conclusions

Blast exposure may result in both overt and subclinical auditory injury in military personnel. While severe blast trauma can cause TMP and mixed hearing loss, individuals with indirect blast exposure may demonstrate subtle cochlear dysfunction despite normal conventional audiograms. Within the scope of this preliminary observation, EHF audiometry and DPOAE testing show promise as potential tools for detecting early cochlear injury and potential HHL following blast exposure. Although validation in larger cohorts is required to establish definitive diagnostic utility, these hypothesis-generating findings may help guide future hearing surveillance strategies for military personnel exposed to blast environments.

## Figures and Tables

**Table 1:** Hearing levels, including standard and extended pure-tone audiogram, in exposed group and control groups.

Age/Sex/Side		Standard Pure-Tone Audiogram (PTA)						Extended High-Frequency PTA				
Exposed group 3–8		250	500	1k	2k	4k	8k	9k	10k	11.2k	12.5k	14k
20/M	R	15	10	10	10	0	5	0	−5	−10	−5	−10
	L	20	15	5	10	5	5	0	−5	−5	0	5
22/M	R	15	15	20	20	20	20	10	5	5	25	10
	L	10	15	15	10	20	10	5	0	−5	10	15
23/M	R	10	10	10	10	20	0	10	10	15	20	50
	L	10	10	15	15	10	5	5	15	15	15	30
29/F	R	10	10	20	10	10	5	5	5	5	10	35
	L	0	5	15	5	5	20	5	5	5	15	25
33/M	R	10	15	10	5	15	20	30	35	40	45	70
	L	15	20	20	15	15	25	30	40	45	50	50
35/F	R	10	5	10	15	10	0	15	15	15	30	70
	L	10	15	15	10	5	0	10	10	15	30	45
Control group 1–6		250	500	1k	2k	4k	8k	9k	10k	11.2k	12.5k	14k
21/F	R	0	0	10	5	5	0	0	0	0	0	0
	L	5	5	0	0	5	0	0	0	0	0	0
24/M	R	0	0	0	0	0	0	0	10	5	0	0
	L	0	0	0	0	0	5	5	0	0	5	5
22/M	R	0	0	0	0	0	0	5	0	10	0	0
	L	0	0	0	0	0	0	0	0	10	5	0
21/M	R	0	0	0	0	5	5	0	5	5	5	0
	L	0	0	5	5	0	0	0	0	0	0	0
22/F	R	5	5	0	0	0	0	0	0	0	0	0
	L	5	0	0	0	0	0	0	5	0	0	10
22/F	R	0	0	0	0	0	0	0	0	0	0	0
E3–8 (Bil, Mean ± SD) *n* = 12		11.25 ± 4.62	12.08 ± 4.31	13.75 ± 4.62	11.25 ± 4.15	11.25 ± 6.50	10.41 ± 8.77	10.42 ± 9.67	10.83 ± 13.51	11.67 ± 16.12	20.42 ± 15.87	32.92 ± 24.19
C1–6 (Bil, Mean ± SD) *n* = 11		1.36 ± 2.23	0.91 ± 1.93	1.36 ± 3.08	0.91 ± 1.93	1.36 ± 2.23	0.91 ± 1.93	0.91 ± 1.93	1.8 ± 3.21	2.73 ± 3.91	1.36 ± 2.23	1.36 ± 3.08
*p*-value		<0.001	<0.001	<0.001	<0.001	<0.001	0.005	0.003	0.017	0.224	0.002	<0.001
E3–8 (R, Mean ± SD) *n* = 6		11.67 ± 2.36	10.83 ± 3.44	13.33 ± 4.71	11.66 ± 4.71	12.5 ± 6.92	8.33 ± 8.50	11.67 ± 9.43	10.83 ± 12.39	11.67 ± 15.18	20.83 ± 15.66	37.5 ± 29.69
C1–6 (R, Mean ± SD) *n* = 6		0.83 ± 1.86	0.83 ± 1.86	1.67 ± 3.73	0.83 ± 1.86	1.67 ± 2.36	0.83 ± 1.86	0.83 ± 1.86	2.5 ± 3.82	3.33 ± 3.73	0.83 ± 1.86	0 ± 0
*p*-value		<0.001	<0.001	0.001	<0.001	0.007	0.082	0.03	0.181	0.2608	0.0176	0.018
E3–8 (L, Mean ± SD) *n* = 6		10.83 ± 6.07	13.33 ± 4.71	14.17 ± 4.49	10.83 ± 3.44	10 ± 5.77	10.83 ± 8.85	9.17 ± 9.75	10.83 ± 14.56	11.67 ± 32.73	20 ± 30.95	28.33 ± 30.28
C1–6 (L, Mean ± SD) *n* = 5		2 ± 2.45	1 ± 2	1 ± 2	1 ± 2	1 ± 2	1 ± 2	1 ± 2	1 ± 2	2 ± 4	2 ± 2.45	3 ± 4
*p*-value		0.021	<0.001	<0.001	<0.001	0.014	0.055	0.13	0.208	0.289	0.051	0.011

**Table 2:** The data of distortion product otoacoustic emissions (DPOAEs) in exposed and control groups.

DPOAE	1k	1.5k	2k	3k	4k	5k	6k	7k	8k	10k
E3–8 (Bil, Mean ± SD) *n* = 12	4.58 ± 4.86	3.62 ± 8.39	5.35 ± 3.18	3.74 ± 3.19	3.21 ± 6.76	3.24 ± 6.03	3.42 ± 6.03	0.99 ± 5.55	2.71 ± 6.99	(−4.3 ± 10.44)
C1–6 (Bil, Mean ± SD) *n* = 11	9.47 ± 4.32	10.78 ± 4.81	8.19 ± 4.07	5.5 ± 3.09	6.2 ± 4.15	7.36 ± 3.52	7.32 ± 3.25	4.76 ± 5.35	2.93 ± 4.55	6.75 ± 7.04
*p*-value	0.024	0.036	0.088	0.214	0.24	0.176	0.082	0.129	0.931	0.01
E3–8 (R, Mean ± SD) *n* = 6	3.43 ± 5.58	4.73 ± 7.17	6.28 ± 3.02	3.43 ± 2.90	2.82 ± 6.41	2.92 ± 5.80	3.4 ± 5.92	0.75 ± 5.62	3.17 ± 8.54	(−3.97 ± 8.47)
C1–6 (R, Mean ± SD) *n* = 6	9.33 ± 3.82	9.58 ± 5.85	7.88 ± 5.40	4.62 ± 3.70	6.5 ± 4.11	8.85 ± 3.38	8.73 ± 2.25	5.6 ± 2.28	3.68 ± 3.35	5.63 ± 6.77
*p*-value	0.091	0.268	0.575	0.585	0.304	0.076	0.104	0.103	0.902	0.075
E3–8 (L, Mean ± SD) *n* = 6	5.73 ± 3.67	2.5 ± 9.32	4.42 ± 3.05	4.05 ± 3.43	3.6 ± 13.6	3.57 ± 6.24	3.43 ± 6.15	1.23 ± 5.47	2.25 ± 4.94	(−4.63 ± 12.09)
C1–5 (L, Mean ± SD) *n* = 5	9.64 ± 4.31	12.22 ± 2.47	8.56 ± 1.09	6.56 ± 1.58	5.84 ± 4.16	5.58 ± 2.78	5.64 ± 3.45	3.76 ± 7.41	2.04 ± 5.53	8.08 ± 7.13
*p*-value	0.175	0.07	0.028	0.205	0.586	0.56	0.534	0.571	0.831	0.093

**Table 3:** The data of auditory brainstem response (ABR) in exposed and control groups.

ABR Latency (ms)	I	III	V	ABR Wave I Amplitude	
E3–8 (Bil, Mean ± SD) *n* = 12	1.60 ± 0.16	3.67 ± 0.14	5.5 ± 0.23	E3–8 (Bil, Mean ± SD) *n* = 12	0.34 ± 0.13
C1–6 (Bil, Mean ± SD) *n* = 11	1.46 ± 0.05	3.55 ± 0.08	5.41 ± 0.08	C1–6 (Bil, Mean ± SD) *n* = 11	0.38 ± 0.11
*p*-value	0.014	0.052	0.052	*p*-value	0.38
E3–8 (R, Mean ± SD) *n* = 6	1.68 ± 0.12	3.68 ± 0.15	5.5 ± 0.1	E3–8 (R, Mean ± SD) *n* = 6	0.27 ± 0.14
C1–C6 (R, Mean ± SD) *n* = 6	1.47 ± 0.04	3.58 ± 0.10	5.42 ± 0.08	C1–6 (R, Mean ± SD) *n* = 6	0.38 ± 0.11
*p*-value	0.005	0.187	0.157	*p*-value	0.215
E3–8 (L, Mean ± SD) *n* = 6	1.52 ± 0.14	3.65 ± 0.17	5.49 ± 0.10	E3–8 (L, Mean ± SD) *n* = 6	0.40 ± 0.07
C1–C5 (L, Mean ± SD) *n* = 5	1.44 ± 0.06	3.52 ± 0.06	5.41 ± 0.07	C1–5 (L, Mean ± SD) *n* = 5	0.38 ± 0.10
*p*-value	0.387	0.183	0.242	*p*-value	0.801

## Data Availability

The datasets analyzed during the current study are available from the corresponding author on reasonable request. The data are not publicly available due to privacy or ethical restrictions..
